# The economic impact of COVID-19: a comparative cost analysis of ICU hospitalizations before and during the pandemic

**DOI:** 10.1016/j.clinsp.2026.100891

**Published:** 2026-03-04

**Authors:** Heloisa Helena Piva, Anna Miethke-Morais, Alex Jones Flores Cassenote, Luiz Augusto Carneiro-D´Albuquerque, Luciana Bertocco de Paiva Haddad

**Affiliations:** Hospital das Clínicas, Faculdade de Medicina, Universidade de São Paulo (HCFMUSP), São Paulo, SP, Brazil

**Keywords:** COVID-19, Intensive care units, Economic evaluation, Cost analysis, Pandemic, Brazil

## Abstract

**Background:**

The COVID-19 economic impact on health institutions has been discussed since the beginning of the pandemic, and the repercussions in different spheres were enormous. The economic evaluation in health became necessary as a management tool.

**Objective:**

To describe and compare the hospitalization costs in Intensive Care Units (ICUs) and the patients' clinical and demographic variables, before and during the COVID-19 pandemic.

**Data sources and study setting:**

Partial economic evaluation of patients admitted to four ICUs, in a Brazilian reference center, in two periods: pre-pandemic (03/30/19 ‒ 06/30/19) and pandemic (03/30/20 ‒ 06/30/20).

**Study design:**

Observational cohort study.

**Data collection/Extraction methods:**

Micro and macro costing methodology; clinical and demographic variables were considered for the comparative analysis.

**Results:**

In 2019, 521 hospitalizations were analyzed. Patients were predominantly male (61%), between 31- and 60-years old (48.2%) and of white race (75.4%). In 2020, 610 hospitalizations were analyzed: 60.3% male, 51.6% > 60-years old and 65.2% white race. All ICUs had an increase in deaths (2019: average of 26.9%; 2020: average of 48.8%). The average total hospitalization costs and the average patient-day cost in 2019 was US$ 6051.01 and US$ 787.48, respectively; in 2020, US$ 19,492.73 and US$ 1542.74. Human resources was the component with the highest cost (2019: 51.6%; 2020: 78.2%). Patients who require additional therapeutic procedures had a higher average cost in 2020.

**Conclusion:**

During COVID, patients were older (> 60-years), a higher percentage of patients identified as mixed race, the death rate was significantly higher, and the length of stay was significantly higher, resulting in the outcome “discharge”. The COVID cost was significantly higher. The main cost component was human resources, mainly during the COVID pandemic. Additional therapeutic procedures were associated with higher costs.

## Highlights

• Average ICU costs tripled during COVID-19 compared to the pre-pandemic period.
• Human resources drove ICU costs, accounting for over 78 % during the pandemic.
• Patients needing ventilation, dialysis or surgery had significantly higher ICU costs.
• The study offers a replicable micro and macro-costing method for economic evaluations.
• Novel comparative analysis of ICU costs before and during the COVID-19 pandemic.

## Background

On December 31, 2019, the World Health Organization (WHO) received notification of several cases of pneumonia in China, in the city of Wuhan, associated with a new type of coronavirus.^1^^,^^2^ One month later, on January 30, 2020, the WHO declared a Public Health Emergency of International Concern.^1^

In Brazil, the confirmation of the first case of COVID-19 occurred on February 26, 2020, in São Paulo, following the Carnival holiday, a period marked by significant movement of foreigners and Brazilians returning from trips abroad.^2^ On March 11, 2020, the WHO declared COVID-19 a pandemic.^1^

Twenty days after the first confirmed case in São Paulo, Brazil, recorded itsthe first death. Within five days, all states had cases and by May 2020, Brazil ranked fourth in cases and sixth in deaths,^3^ reaching 7.68 million cases and 194,949 deaths by the end of 2020.^4^

In the world, in May 2020, there were 4425,485 cases of COVID-19 and 302,059 deaths, across 216 countries^3^ and, by the end of 2020, there were 83.43 million confirmed cases and 1.82 million deaths.^4^

2021 was marked by the second wave of the coronavirus, with a significant impact on mortality. On January 17, 2021, the Brazilian Health Regulatory Agency (Agência Nacional de Vigilância Sanitária ‒ ANVISA) approved the emergency use of the CoronaVac vaccine. The increase in vaccine coverage reduced the number of cases by 80.5 % and deaths by 94.9 %.^5^

Brazil ended the year 2021 with 22,287,521 confirmed cases and 619,056 deaths.^6^ Until December 31, 2023, there were 6991,842 deaths worldwide^7^ and 708,638 in Brazil.^6^

Hospital das Clínicas of the University of São Paulo School of Medicine (Hospital das Clínicas da Faculdade de Medicina da Universidade de São Paulo ‒ HCFMUSP) launched the Crisis Committee in January 20,20^8^^,^^9^ to organize the efforts, including the development of medical protocols for patient treatment and preparation hospital to receive patients from the public healthcare system.

To organize patient care for those diagnosed with COVID-19 and ensure the continuation of treatment for other patients requiring tertiary hospital care, in March 2020 was decided to isolate the Central Institute (Instituto Central do Hospital das Clínicas ‒ ICHC) for the exclusive treatment of COVID-19.^2^^,^^8^

Other institutes were mobilized to continue attending to the non-COVID patient demand. All patients were transferred to the other institutes and the ICHC became a reference center for treating severe COVID-19 patients. This patient care demand was only possible to be absorbed through an agreement made with the State Department of Health of São Paulo (Secretária de Estado de Saúde de São Paulo ‒ SES).

Regarding physical structure, there was an unquestionable need to expand the number of ICU beds. Five to 20 % of COVID-19 patients require hospitalization, of which 14 % to 20 % need intensive care for disease treatment during hospitalization due to developing severe hypoxemia requiring ventilatory support, among other complications.^10^, ^11^, ^12^, ^13^, ^14^

In response, the Brazilian Ministry of Health (Ministério da Saúde ‒ MS) authorized the temporary qualification of Adult Intensive Care Unit beds for exclusive COVID-19 patient care.^15^ Initially, the expansion was from 84 to 200 beds. Two months later, an additional 100 beds were made available, totaling 300 ICU beds and 500 ward beds. Operating rooms and wards were converted into ICU beds.^16^^,^^17^

Part of the teams from all specialties were reassigned to other institutes; part remained in the ICHC for COVID-19 patient care.^2^ The agreement with the SES also enabled the hiring of temporary professionals, in addition to donations that allowed additional hirings.

In September 2020, with the beginning of the regression in the number of cases, the ICHC was demobilized and specialties gradually returned to the institute. At this moment, the ICHC ceased to be the only institute to receive COVID-19 patients in the HCFMUSP.

Given the significant repercussions caused by COVID-19, including economic impact,^18^ it is necessary to understand the extent of this impact. Health Economic Evaluation (HEE) is a branch of Health Technology Assessment (HTA) that measures the cost dimension of the evaluated resources and their consequences. The goal of such evaluation is to standardize the structuring of information to provide reliable data that can support decision-making by prioritizing and optimizing resources.^19^^,^^20^

A study on economic evaluation in COVID-19, with a sample of 3254 patients with suspected or confirmed disease, showed that on average, the cost of hospitalization for patients was $12,637.42, with a daily average cost of $919.24. Patients needing intensive care during hospitalization cost an average of $20,002.80. Others, who did not require ICU support, cost an average of $4839.57.^11^

Patients with confirmed COVID-19 had higher average costs, as did patients with comorbidities (the more comorbidities, the higher the average cost) and those requiring procedures during hospitalization (mechanical ventilation, tracheostomy, hemodialysis and surgeries).^11^

In terms of cost components, the care team, medical and non-medical, represented the highest cost (82 % of the total). Other components with higher costs include medications, materials and laboratory tests in the ICU, personal protective equipment, medications and materials in wards and laboratory tests, radiological exams and medications in emergency units.^11^

Studies on HEE like this provide important information for healthcare managers' decision-making.^20^

Given the global impact of the pandemic, including its economic ramifications, the complexity of healthcare delivery, the need for healthcare service restructuring, and the magnitude of the HCFMUSP and its role as a reference service for COVID-19 treatment, the justification for this study lies in providing essential information for healthcare managers across various spheres and perspectives.

Through this study, it is possible to understand and compare costs between different periods and across different ICU settings, as well as how costs were distributed among various components. Additionally, it aims to identify clinical factors directly impacting the healthcare service's economic scenario during both periods.

The main objective of this study was to describe and compare the cost of hospitalizations in four Intensive Care Units (ICUs) within the ICHC before and during the pandemic. Furthermore, it seeks to differentiate the economic impact by ICU and clinical and demographic variables.

## Methods

### Sample

This is an observational cohort study, conducted in accordance with the STROBE Statement - Strengthening the Reporting of Observational Studies in Epidemiology, of a partial economic evaluation of patients admitted to four ICUs at the ICHC. The perspective used was that of the hospital, the healthcare service provider.

To achieve the study's objectives, four ICUs from the ICHC were selected: Infectious Diseases and Clinical Emergencies ICUs (clinical profile) and Trauma and Gastroenterology ICUs (surgical profile). The admission profile refers to the pre-pandemic period.

The study population consisted of patients admitted to the four selected ICUs in two periods: March 30, 2019, to June 30, 2019, considered the pre-pandemic period, and March 30, 2020, to June 30, 2020, considered the pandemic period.

In 2019, the selected ICUs admitted patients with either clinical or surgical profiles. In 2020, the care provided was exclusively focused on patients suspected or confirmed to have COVID-19.

The research protocol was approved by the institutional ethics committee (CAPPESQ: n° 4.337.320). The requirement for obtaining informed consent was formally waived, given that the study was based on retrospective data obtained from an existing database.

The clinical and demographic variables used were gender, age, race, length of stay, procedures (mechanical ventilation, dialysis, and surgeries), and clinical outcome (discharge, death or transfer).

This information was extracted from the MV system ‒ Electronic Health Record (EHR), comprising both the electronic medical record (clinical records, progress notes, and prescriptions) and the administrative part (patient movement, dispensing of medications and materials).

Race data were obtained from the MV system, extracted from the patient identification fields, which were completed by the administrative staff at the time of admission, based on the patient's self-identification or, when not possible, on a family member's report.

### Measurement of cost

Direct costs included medications, laboratory tests, radiological exams, enteral and parenteral diets and blood components. These are part of the prescription items in the electronic system and were therefore extracted from the patient's electronic medical record.

For medications and enteral and parenteral diets, all prescribed and checked items were considered, and the costs refer to the competence period, also extracted from the MV system.

Regarding laboratory tests, the prescribed items in the MV system were considered. However, the cost of the tests is not registered in the database of the computerized system. The attributed cost, for 2020, was calculated and provided by the Central Laboratory Division (Divisão de Laboratório Central ‒ DLC).

For the cost of radiological exams, also prescribed in MV, a survey conducted in 2020 with the support of the Cardiology Institute (Instituto do Coração ‒ InCor) and the Radiology Institute (Instituto de Radiologia ‒ InRad) was considered.

For blood components, all administered to the patients in the sample were considered, and for the cost, a survey of the value conducted in 2020 with the support of the InCor was considered.

To compose the cost of patient hospitalization in the ICUs, added to the direct costs, information from the cost model database implemented at the ICHC was used, organized by cost centers. Each cost center receives all costs related to the operation of the structure/unit, considering indirect costs and allocations.

Cost groups make up the value of the cost center/month, grouped as follows: general costs (water and sewage, electricity, insuranceand telephony), general consumer materials (other materials, personal protective equipment, office materials, hygiene materials, maintenance materials and uniforms/linens), patient-use materials (medications, medical supplies, prostheses, laboratory use materials), human resources (medical and non-medical) and service provision.

To compose the sector's cost calculation, all the above items were considered, except for medications and Human Resources (HR), as both have particularities described below:

• Medications were analyzed per patient and, therefore, attributed a direct cost.
• Human Resources (HR), medical and non-medical, were treated outside the sector's cost.

The cost of the professionals who worked in 2019 was allocated to the cost center of the unit in which each professional carried out their activities. Thus, the cost of each team, considering the cost of every professional within it, was allocated to this cost center, which served as the data source for 2019. In 2020, professionals were allocated to a single cost center. Therefore, for cost calculation, the staffing levels of each unit were considered, and the cost of each professional, including that of volunteer professionals, was assigned accordingly. The volunteers were professionals from other hospitals who continued to be paid by their home institutions. However, for calculation purposes in this study, the remuneration corresponding to their position at the institution was considered, as if these professionals had been hired directly.

General costs are valued according to the size/infrastructure of the unit. General consumer materials and patient consumption materials are requested for the unit directly by the cost center and are not dispensed to patients. Thus, they are included in the general value of the cost center. Service provision contracts are also measured through the cost center.

With the composition of the cost related to the unit's operation, each cost center arrives at the total primary cost value. Added to this cost are the allocations, which refer to the costs of support units for assistance (for example, clinical engineering, hospitality, janitorial services, supplies, property security etc.).

After the inclusion of allocations, the total sector cost is calculated, monthly production is quantified, and the unit's patient-day cost is determined.

To compose the cost of patients admitted to the study ICUs, the patient-day cost proportional to the patient's length of stay in the unit/cost center was considered, considering the ICU/internship itinerary (daily patient-day).

Thus, the total cost was composed of direct costs (medications, radiological exams, laboratory tests, blood components and diet) and indirect costs (allocations, materials, general costs, human resources, services, contracts and daily rates), according to the patient's itinerary.

Continuous variables were expressed as mean ± standard deviation, and categorical variables as number of cases and proportions. To assess the impact of different variables on hospital costs, the length of stay of each subgroup of patients was considered.

The average cost per admission refers to the total average cost of admission for each patient and the patient-day cost refers to the total cost/total follow-up in days.

Cost data were collected and analyzed in Brazilian currency (reais). The dollar was calculated based on the average value of the currency during the study period. For the 2019 sample (from 03/30 to 06/30): 1 dollar = 3.92 reais; for the 2020 sample (from 03/30 to 06/30): 1 dollar = 5.37 reais.^21^

For the discussion, the same conversion method was adopted; costs that were not in dollars were converted considering the average value of the currency during the study period for comparison with the results found.

### Statistical analysis

The hypothesis testing applied considered an alpha error of 0.05. Continuous variables were compared between groups using the Kruskal-Wallis test, and categorical variables were compared using the Chi-Square test or Fisher's exact test. To obtain the adjusted impact of the variables of interest on total cost, a Generalized Linear Model (GLM) with gamma probability distribution and log-link function was proposed. The GLM was adjusted for the following covariates: sex, age, invasive mechanical ventilation, dialysis, surgery, outcomes, ICU admission and length of hospital stay. Statistical analyses were performed using IBM SPSS Statistics v. 26.0 (SPSS Inc., Chicago, Illinois, USA).

## Results

### Study population

Between March 30 and June 30, 2019, 521 admissions were conducted in the four selected ICUs, with 61 % of the patients being male, predominantly (48.2 %) aged between 31 and 60-years, and 75.4 % of the white race. In the same period, in 2020, and in the same ICUs, 610 admissions were conducted, with 60.3 % of the patients being male, 51.6 % aged over 60-years, and 65.2 % of the white race (Table 1).

**Table 1:** Distribution of hospitalizations by gender, age and race in 2019 and 2020.

Variables		2019		2020		p-value^a^
		n	%	n	%	
Gender	Female	203	39.0 %	242	39.7 %	0.047
	Male	318	61.0 %	368	60.3 %	
Age	≤ 30 years old	67	12.9 %	32	5.2 %	<0.001
	31 ‒ 60 years old	251	48.2 %	263	43.1 %	
	> 60 years old	203	39.0 %	315	51.6 %	
Race	Yellow	6	1.2 %	2	0.3 %	0.017
	White	393	75.4 %	398	65.2 %	
	Brown	52	10.0 %	153	25.1 %	
	Black	42	8.1 %	41	6.7 %	
	No information	28	5.4 %	16	2.6 %	

a Chi-Square test. Refers to the comparison of variables between periods.

### Outcome

All ICUs experienced an increase in the number of deaths when comparing the samples from 2019 to 2020. Trauma ICU increased from 32 % in 2019 to 50.8 % in 2020; Gastroenterology ICU from 25.7 % to 55.1 %; Infectious Diseases ICU from 18.9 % to 48 %; and Clinical Emergencies ICU from 31 % to 41.4 %.

**Fig. 1:**
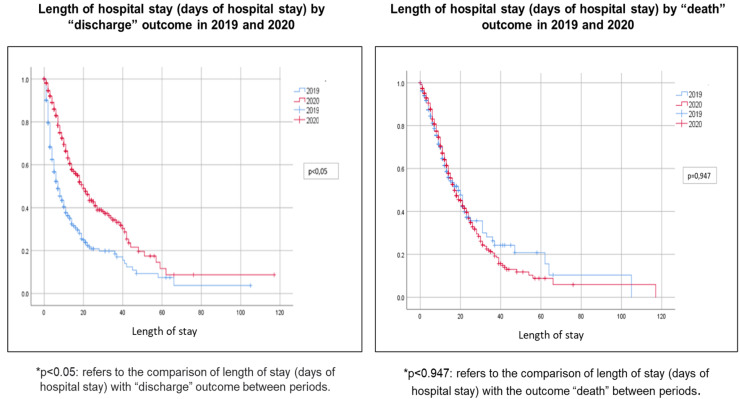
Length of hospital stay by outcome in 2019 and 2020.

Regarding the outcome, the length of stay for patients who were discharged was significantly longer in 2020 compared to 2019 (*p* < 0.05). However, concerning death, there was no significant difference in the length of stay when comparing 2019 with 2020 (Fig. 1).

### Cost

**Fig. 2:**
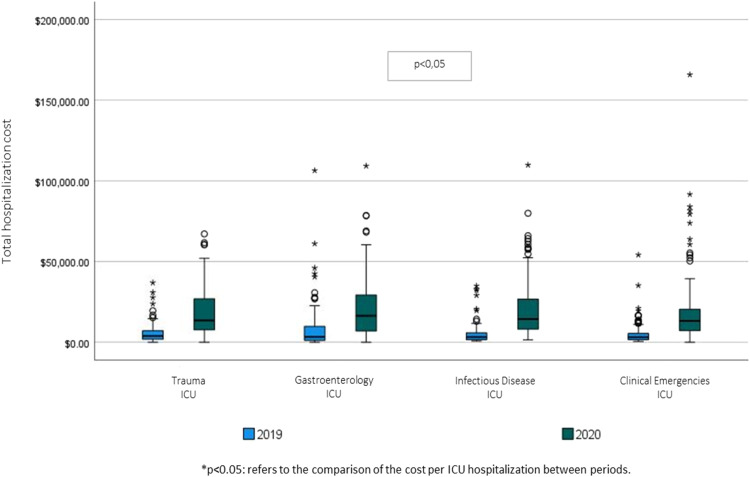
Total hospitalization cost per ICU in 2019 and 2020.

The total cost per admission and the cost per patient-day were higher in all ICUs when comparing the 2019 sample with that of 2020 (Fig. 2).

In 2019, the ICU with the highest cost per admission and per patient-day was the Gastroenterology ICU (US$ 8090.66 and US$ 1072.23, respectively) and it remained as the ICU with the highest cost in 2020 (US$ 21,797.19 and US$ 1672.70) (Fig. 2).

The average total cost of ICU admissions in 2019 was US$ 6051.01 and the average cost per patient-day was US$ 787.48. In 2020, during the COVID period, the average cost increased significantly to US$ 19,492.73 (cost per admission) and US$ 1542.74 (cost per patient-day).

Regarding cost components, the indirect cost of non-medical and medical human resources was the component with the highest cost for all ICUs, both in 2019 and 2020, representing on average 51.6 % and 78.2 %, respectively. However, this percentage was significantly higher in the 2020 period (*p* < 0.001).

Another important cost component was the sector, which represented on average 29.6 % in 2019 and 15.88 % in 2020 (Table 2).

**Table 2:** Distribution of the hospitalizations cost by component and ICU in 2019 and 2020.

ICU	Cost Componente	2019		2020		GLM p-value^a^
		Mean (US$)	Standard deviation (US$)	Mean (US$)	Standard deviation (US$)	
**Trauma ICU**						
	Sector	1588.84	1647.49	3186.42	2578.61	<0.001
	Human resources	3344.38	3431.48	14,776.27	11,937.98	
	Blood componentes	228.78	313.79	175.23	153.55	
	Radiologic exams	132.87	177.59	52.05	65.26	
	Laboratory tests	154.91	168.82	253.14	198.28	
	Drugs	232.01	329.80	593.92	680.23	
**Gastroenterology ICU**						
	Sector	2552.58	4182.09	4722.99	4290.67	<0.001
	Human resources	3856.50	6368.69	15,819.09	14,172.74	
	Blood componentes	683.09	1295.24	196.01	198.58	
	Radiologic exams	96.98	186.99	39.64	48.55	
	Laboratory tests	320.35	532.42	407.06	373.34	
	Drugs	1020.37	2004.35	763.01	1710.60	
**Infectious Disease ICU**						
	Sector	2331.53	3031.90	2472.85	2144.28	<0.001
	Human resources	2444.14	3175.83	15,999.88	13,884.94	
	Blood componentes	279.18	323.20	241.63	305.87	
	Radiologic exams	96.55	141.54	57.45	57.37	
	Laboratory tests	242.06	344.53	359.12	311.99	
	Drugs	593.26	1837.83	831.46	1193.87	
**Clinical Emergencies ICU**						
	Sector	1074.05	1214.32	2278.17	2544.77	<0.001
	Human resources	3089.08	3498.61	14,627.89	16,084.74	
	Blood componentes	195.22	143.63	143.68	100.24	
	Radiologic exams	82.95	130.01	38.60	52.05	
	Laboratory tests	129.73	168.24	189.20	154.26	
	Drugs	421.65	1955.32	380.37	487.80	

a p, Generalized Linear Model (GLM) with gamma probability distribution and log link function. Refers to the cost of each component per ICU between periods. The model was adjusted for sex, age, Invasive Mechanical Ventilation (IMV), dialysis, surgery, outcomes, ICU admission, and length of hospital stay.

Additional therapeutic procedures during admission (mechanical ventilation, dialysis and surgery) were also significantly associated with higher total and daily average costs in 2020 compared to 2019 (Table 3).

**Table 3:** Distribution of the cost of hospitalizations according to the procedures performed in 2019 and 2020.

Procedures		2019		2020		GLM p-value^a^
		Mean (US$)	Standard deviation (US$)	Mean (US$)	Standard deviation (US$)	
**Mechanical ventilation**						
Cost per patient	No	3022.52	3334.92	7006.44	5733.50	<0.001
	Between 1‒5 days	4483.98	4342.31	9974.26	9027.75	
	> 5 days	15,306.55	14,170.46	28,542.67	19,093.92	
Patient-day cost	No	772.86	289.04	1480.64	75.20	
	Between 1‒5 days	753.52	303.08	1538.00	201.61	
	> 5 days	765.22	269.48	1540.95	99.30	
**Dialysis**						
Cost per patient	No	5178.48	6694.83	16,536.33	16,845.83	0.005
	Yes	10,110.49	14,115.35	25,371.23	18,780.81	
Patient-day cost	No	748.13	259.32	1502.23	85.42	
	Yes	844.42	398.72	1585.79	189.73	
**Surgery**						
Cost per patient	No	4659.71	5762.41	18,494.15	16,216.89	<0.001
	Yes	14,177.19	15,665.66	35,477.27	36,096.67	
Patient-day cost	No	756.55	292.19	1528.46	134.84	
	Yes	812.48	271.77	1526.68	94.24	

a p, Generalized Linear Model (GLM) with gamma probability distribution and log link function. Refers to the cost of each component per ICU between periods. The model was adjusted for sex, age, Invasive Mechanical Ventilation (IMV), dialysis, surgery, outcomes, ICU admission, and length of hospital stay.

Patients who did not require mechanical ventilation had lower admission costs (2019: US$ 3022.50 and 2020: US$ 7006.44) compared to patients who required between 1- and 5-days of MV (2019: US$ 4483.98 and 2020: US$ 9974.26) and >5-days of MV (2019: US$ 15,306.55 and 2020: US$ 28,542.67). The increase for the 2020 period was also observed in other procedures (dialysis and surgery) (Table 3).

## Discussion

As described by Clarke,^20^ health economic evaluation provides important insights for decision-making by managers at various levels. Considering the COVID-19 pandemic and its widespread repercussions, including economic ones, understanding costs and their distribution within healthcare services enables planning for future similar situations; therefore, it is the first aspect of great relevance in this study.

All studies focus solely on costs during the pandemic, from various perspectives, related to hospitalized patient care.^12^^,^^22^^–^^33^ However, comparing costs, from the hospital perspective and in detail (per patient, patient-day, component, outcome and procedures performed), between pre-pandemic and pandemic care is the second aspect of relevance and novelty in this study. No other studies were found comparing hospitalization costs between the two periods within the same institution.

More than understanding costs, comparing them to pre-pandemic situations enables the understanding of the concrete and objective need for financial support to address such situations.

As described above, most studies focus solely on costs during the pandemic^22^, ^23^, ^24^, ^25^, ^26^, ^27^, ^28^, ^29^, ^30^, ^31^, ^32^ and do not compare cost outcomes with another period.

The provider/service perspective, used in the present study, was also observed as the most commonly used in other studies.^11^^,^^22^^,^^23^^,^^25^^,^^27^^–^^29^^,^^31^^,^^32^ However, there are also studies that evaluated from other perspectives: healthcare system, federation, payer and society.^12^^,^^22^^,^^24^^,^^26^, ^27^, ^28^, ^29^, ^30^^,^^33^

Evaluation from various perspectives allows for relevant discussions and for planning and management across different spheres involved in healthcare provision, whether as resource providers, service providers, or society.

The costing methodology used in all studies was micro and macro-costing. However, even with the same methodology, there is diversity in the components considered. Only a few studies consider, for example, the partial or total cost of the care team^11^^,^^22^^,^^23^^,^^28^, ^29^, ^30^, ^31^, ^32^, ^33^ and costs of diagnostic tests.^11^^,^^12^^,^^28^, ^29^, ^30^, ^31^, ^32^, ^33^

Regarding patients' admission locations, all studies considered all sectors where patients were assisted (emergency units, wards, and ICUs). ^,^^11^ 22, 23, 24, 25, 26, 27, 28, 29, 30, 31, 32, 33

The only study presenting a comparative analysis of costs between the pre-pandemic period and the pandemic period is the study by Nascimento et al.,^26^ which uses the federation perspective (Brazil). However, it does not describe the cost of hospitalizations but rather the total public spending on healthcare services, outside the scope of discussion of this study.

Due to the heterogeneity of methods (perspective, economic evaluation methodology, patient profile, among others) and the limited references comparing costs between COVID and non-COVID periods, discussing the cost results of the samples is challenging.

The average cost of COVID hospitalizations in this study was US$ 19,492.73 (R$ 104,675.98). In other studies,^11^^,^^12^^,^^22^, ^23^, ^24^, ^25^^,^^27^, ^28^, ^29^, ^30^, ^31^, ^32^, ^33^ the cost ranged from US$ 1385.80^27^ to US$ 20,002.80.^11^ The variation can be explained by the evaluated cost perspective and the considered cost components. Specificities of some studies will be discussed below.

In a recent study, Cardoso et al.^23^ analyzed the costs of COVID-19 patients according to their hospital trajectory, with averages ranging from US$ 872.32 (patients not admitted to the ICU) to US$ 7317.90 (patients in the ICU). The difference compared to this study is due to the cost components analyzed, as materials, diets, and non-medical teams were not included. The most significant cost component in both studies remains human resources (74 %).

Miethke-Morais et al.^11^ highlighted an average hospitalization cost of US$ 12,637.42 in the same institution as this study. However, the authors considered the complete patient care trajectory, including the emergency unit, ward, and ICU. When considering only patients who were admitted to the ICU during hospitalization, the average cost of hospitalization is US$ 20,002.80, very similar to that of this study.

The same study^11^ also identified human resources as the main cost component, as well as higher average costs in patients who underwent surgical procedures, dialysis, or mechanical ventilation, corroborating the results of this study.

It is important to highlight that the proportional increase in human resource costs in 2020 reflects the rise in the number of professionals hired at the ICHC to meet the expansion of ICU beds during the COVID-19 pandemic. There were no payments for overtime, hazard pay, or other salary supplements to professionals during the analyzed period.

Regarding procedures, Xavier et al.^22^ reported increased costs in patients receiving mechanical ventilation, consistent with the findings of this study. Khan et al.^12^ also observed higher costs for patients on mechanical ventilation in both the ward and ICU. Additionally, Oliveira et al.^27^ identified dialysis as another factor contributing to increased costs.

Regarding the year 2019, the non-COVID period, the average hospitalization cost in this study was US$ 6051.01 (R$ 23,719.97). No other studies were found that compared costs between the periods; therefore, the discussion is limited to studies on the cost of ICU-hospitalized patients in a care pathway.

Studies describe the cost of patients with hospital-acquired infections hospitalized in intensive care units.^34^, ^35^, ^36^, ^37^, ^38^ Izaias et al.^37^ described antibiotic therapy as responsible for 5 % of hospitalization costs. Both Lara et al.^34^ and Nangino et al.^35^ reported the impact of hospital-acquired infections and antimicrobial use on hospital costs, both due to the cost of the medication itself and its consequences: increased average length of stay, mortality, and bacterial resistance.

Considering this scenario, Reis et al.^36^ conducted a literature review and discussed the importance of early mobilization of critically ill patients to facilitate weaning from mechanical ventilation, with future complications, including hospital-acquired infections and antibiotic therapy, responsible for the highest costs of hospitalizations.

Hospitalization costs in non-COVID studies ranged from US$ 1514.79^34^ and US$ 17,359.30.^38^ However, the samples consisted of patients with hospital-acquired infections and the considered components were quite diverse; in Lara et al. study,^34^ for example, the cost referred only to antibiotic therapy. Therefore, these studies are not comparable, highlighting the relevance of the present study.

It is worth noting that, beyond the economic aspects, the high mortality observed reflects the severity of COVID-19 and the challenges faced by the healthcare system. The need for preparedness plans, support for healthcare teams, and public health measures to mitigate avoidable mortality and its social impacts becomes essential during such periods.

The preparation of HCFMUSP for the crisis was made possible by several factors, including the structure of the Brazilian Unified Health System (SUS), the agreement with the State Health Department (SES) and the hospital’s rapid response capacity, supported by the physical infrastructure of its various institutes and previous experience in managing other health crises. In different contexts, the lack of support, infrastructure, and experience may represent limitations for large-scale and rapid adaptations.

The urgent need to expand ICU bed capacity and, consequently, the healthcare workforce, highlights the importance of public health emergency preparedness strategies focused on resource expansion, clinical management protocols, professional training and prevention policies. This framework is a key component of resilience strategies for pandemics and public health crises.

This study has some limitations. First, there was a change in the data source used to calculate human resources in 2019 and 2020, which may have introduced some variability in the estimates. Second, the average exchange rate for the periods was used, common in retrospective economic analyses, but it does not capture short-term volatility; monthly or daily rates could provide more precise estimates. Third, there was no adjustment for disease severity between cohorts; differences in age, mortality and baseline severity may have contributed to part of the increase in ICU costs, beyond the effect of COVID-19.

## Conclusion

During the COVID period, patients were older (> 60-years), there was a higher percentage of brown, the mortality rate was significantly higher and the length of stay in the ICU was significantly longer for patients whose outcome was hospital discharge. COVID-related costs were significantly higher than costs in the non-COVID period. The main cost component was human resources and additional therapeutic procedures were also associated with higher total and daily average costs during COVID.

## Authors' contributions

Heloisa Helena Piva: Conceptualization; methodology; investigation; validation; data curation; writing-original draft; writing-review & editing; visualization.

Anna Miethke Morais: Conceptualization; methodology; investigation; validation.

Alex Jones Flores Cassenote: Conceptualization; software; formal analysis; data curation; resources.

Luiz Augusto Carneiro D´Albuquerque: Conceptualization; project administration.

Luciana Bertocco de Paiva Haddad: Conceptualization; methodology; investigation; validation; writing-review & editing; supervision; project administration.

## Data availability

The datasets generated and/or analyzed during the current study are available from the corresponding author upon reasonable request.

## Conflicts of interest

The authors declare no conflicts of interest.
